# Effects of Hydration Level and Heat Stress on Thermoregulatory Responses, Hematological and Blood Rheological Properties in Growing Pigs

**DOI:** 10.1371/journal.pone.0102537

**Published:** 2014-07-11

**Authors:** Xavier Waltz, Michelle Baillot, Philippe Connes, Bruno Bocage, David Renaudeau

**Affiliations:** 1 UMR Inserm 1134, Pointe-à-Pitre, F-97159 Guadeloupe, Université des Antilles et de la Guyane, Pointe-à-Pitre, France; 2 Laboratoire ACTES (EA 3596), Département de Physiologie, Université des Antilles et de la Guyane, Pointe-à-Pitre, Guadeloupe, France; 3 Laboratory of Excellence GR-Ex, PRES Sorbonne Paris Cité, Paris, France; 4 Institut Universitaire de France, Paris, France; 5 INRA, UR 143, Unité de Recherches Zootechniques (URZ), Petit Bourg, France; 6 INRA, UMR 1348, Physiologie, Environnement et Génétique pour l'Animal et les Systèmes d'Elevage (PEGASE), Rennes, France; INIA, Spain

## Abstract

Heat stress is one of the major limiting factors of production efficiency in the swine industry. The aims of the present study were 1) to observe if hemorheological and hematological parameters could be associated to physiological acclimation during the first days of heat stress exposure and 2) to determine if water restriction could modulate the effect of thermal heat stress on physiological, hematological and hemorheological parameters. Twelve Large White male pigs were divided into an *ad libitum* and a water restricted group. All pigs were submitted to one week at 24°C (D-7 to D-1). Then, at D0, temperature was progressively increased until 32°C and maintained during one week (D1 to D7). We performed daily measurements of water and feed intake. Physiological (i.e., skin temperature, rectal temperature, respiratory rate), hematological and hemorheological parameters were measured on D-6, D-5, D0, D1, D2 and D7. Water restriction had no effect on physiological, hematological and hemorheological parameters. The first days of heat stress caused an increase in the three physiological parameters followed by a reduction of these parameters suggesting a successful acclimation of pigs to heat stress. We showed an increase in hematocrit, red blood cell aggregation and red blood cell aggregation strength during heat stress. Further, we observed an important release of reticulocytes, an increase of red blood cell deformability and a reduction of feed intake and blood viscosity under heat stress. This study suggests that physiological acute adaptation to heat stress is accompanied by large hematological and hemorheological changes.

## Introduction

Heat stress is one of the major limiting factors of production efficiency in the swine industry by reducing voluntary feed intake with subsequent negative consequences on growth performance [1], [2]. While thermal stress is an occasional problem in temperate areas during the 2–3 summer months, it is a chronic issue in many tropical countries where pig production has grown at a high rate during the last two decades [1].

Blood rheology is a key factor of blood flow and cardiovascular functioning [3], [4] and hemorheological changes under heat stress could interfere with the physiological thermoregulatory adaptations [5]. We recently investigated the effect of heat stress on physiological, hematological, hemorheological parameters in different pig breeds [5]. In this experiment, three different pig breeds with assumed different heat stress tolerance were studied: Large White (little tolerance), Creole (good tolerance) and a cross between Large White and Creole breeds. Although red blood cell deformability was higher in Creole pigs exposed to heat stress compared to Large White pigs, the hematological and hemorheological responses to heat stress were very similar [5]. However, in this previous study, the physiological, hematological and hemorheological parameters were measured after 5 days of heat stress exposure and pigs were probably acclimatized at that time. It was thus not possible to compare the ability of pigs to adapt to heat stress. To the best of our knowledge, the time for hematological and hemorheological parameters to normalize under heat exposure is unknown in pigs and might be connected to animal thermoregulatory responses.

The aims of the present study were 1) to observe if hemorheological and hematological changes could be associated to physiological acclimation during the first days of heat stress exposure and 2) to determine if water restriction could modulate the effect of thermal heat stress on physiological, hematological and hemorheological parameters. We hypothesized that short-term physiological adaptation to heat stress could be accompanied by hematological and hemorheological changes. In addition, we hypothesized that the lack of changes in hematological and hemorheological parameters occurring after 5 days of heat stress exposure [5] could be explained by the changes in water consumption occurring immediately after the beginning of heat stress exposure. Therefore, water restriction should affect hematological and hemorheological changes and physiological acclimation during heat stress exposure. We compared physiological, hematological and hemorheological responses during a 7-day thermal acclimation period at 32°C between a water restricted and an *ad libitum* group of growing pigs.

## Materials and Methods

### Experimental design and animal management

This study was performed at the experimental facilities of INRA Guadeloupe, French West Indies. These facilities have a certificate of authorization to experiment on living animals issued by the French ministry of Agriculture (number: A 971-18-02). Care and use of animals were performed in strict accordance with the recommendation in the guide for the care and use of laboratory Animals of the French national institute of health. A total of 12 Large White male pigs were used in this experiment. At 70 days of age (31.4±2.6 kg), pigs were selected according to their litter origin (n = 6), body weight and general sanitary status and moved to a climate-controlled room. Practically, 2 pigs with a similar body weight were selected within each litter and allowed to one of the two water treatments (*ad libitum* vs. 70% of *ad libitum* voluntary water intake: called “water restricted group”). Within each litter, water allowance of the water restricted pigs was calculated every day from the water intake of the littermate in the *ad libitum* group the day before. Pigs were adapted to the experimental conditions (housing, diet, water restriction) for 8 days. During the adaptation period, ambient temperature was maintained at 24°C. Then, the 14 days of experimental period were divided in two consecutive phases of 7 days each. First, pigs were maintained at 24°C for 7 days (D-7 to D-1) and were kept thereafter at a constant temperature of 32°C for 7 days (D1 to D7). Between these two phases, ambient temperature was changed on day 0 (D0) from 10:00 h to 14:00 h at a constant rate of 2°C/h. The relative humidity was maintained constant at 80% over the total duration of the experiment and the photoperiod was fixed to 12:00 h artificial light (from 06:00 a.m. to 06:00 p.m.). Pigs were individually housed in metal slatted pens (2.0×1.0 m). Each pen was equipped with a feed dispenser and a nipple drinker designed to avoid water spillage and connected to a 20 L water tank. For the water restricted group, the water tanks were filled up twice a day (i.e., at 09:00 and 12:00). Pigs were fed *ad libitum* with a commercial diet. This diet was formulated from corn and soybean meal to cover their energy and the protein requirements.

### Physiological measurements

Pigs were weighted at fixed hour (i.e., 08:00 h) on D-8, D0 and D8. Feed intake was recorded as the difference between food allowance and refusals collected the following morning. The water tanks were filled twice a day at 08:00 and 12:00. Similarly to feed, water consumption was determined as the difference between water allowance and refusals and spillages collected the following morning. Feed and water intake were recorded each day from D-6 to D7.

Rectal and skin body temperatures and respiratory rate were measured at 11:00 h on D-6, D-5, D0, D1, D2 and D7. The respiratory rate (breaths per minute) was determined by counting flank movements only in resting animals for a period of 1 min. In practice, two measurements were performed by two different persons. The ability of pigs to sweat is limited and heat losses mainly occur by respiratory evaporation. Therefore, increased respiratory rate in hot conditions participate to the loss of water in pigs [6]. After respiratory rate measurements were completed, skin temperature was measured on the back by using a digital thermometer (HH-21 Model, Omega, Stamford, CT) with a K contact probe. Variation in skin temperature under heat stress conditions is an indicator of increased blood flow to the skin to promote sensible heat loss [7]. Rectal temperature was measured by using a digital medical thermometer.

### Hematological and hemorheological measurements

Blood samples were taken by a direct venipuncture on the jugular vein at 10:00 h on D-6, D-5, D0, D1, D2 and D7. Blood was collected into 2×5 mL heparinized and 1×5 mL EDTA tubes and samples were stored at 4°C before hemorheological and hematological measurements. In practice, pigs were restrained on their back by two handlers while a third experimenter took blood samples. To avoid excessive stress on the animal, this procedure never exceeded 2 minutes [5].

Hemoglobin concentration, hematocrit, mean cell volume, mean cell hemoglobin concentration, percentage of reticulocytes, red blood cells (RBCs), white blood cells and platelets counts were determined using a hematology analyzer (Max M-Retic, Coulter, USA).

All hemorheological measurements were carried out within 4 hours of blood sampling to avoid blood rheological alterations [8], and after complete oxygenation of blood [9]. We strictly followed the international guidelines for the standardization in blood rheology techniques/measurements [9]. Blood viscosity was determined at native hematocrit, at 25*°*C and at 225 s*^−^* ^1^ using a cone/plate viscometer (Brookfield DVII+ with CPE40 spindle, Brookfield Engineering Labs, Natick, MA). RBC deformability (Elongation Index; EI) was determined at 37°C at two shear stresses (3 and 30 Pa) by laser diffraction analysis (ecktacytometry), using the Laser-assisted Optical Rotational Cell Analyzer (LORCA, RR Mechatronics, Hoorn, The Netherlands). RBC aggregation was determined at 37°C via syllectometry, (i.e., laser backscatter versus time, using the LORCA) after adjustment of the hematocrit to 40%, and was reported as the aggregation index. The RBC disaggregation threshold, i.e., the minimal shear rate needed to prevent RBC aggregation or to break down existing RBC aggregates, was determined using a re-iteration procedure [10].

### Statistical analysis

The Proc Mixed model (SAS Inst. Inc., Cary, NC) was used to test the effects of temperature and hydration level on all response variables. Daily feed and water intakes (14 measurements/animal) were analyzed with a model including the effects of temperature (2 levels), water restriction (2 levels), days of exposure within temperature effect (14 levels), and interactions. The model had a repeated structure for day of exposure, which allowed the incorporation of heterogeneity of variances across days. Contrasts were generated to compare data recorded from D0 to D7 (i.e., 32°C) to average value of data between D-6 and D-1 (i.e., thermoneutral condition considered as a reference value). Thermoregulation, hematological and hemorheological parameters (6 measurements/animal) were analyzed with a similar previous model. When a day of exposure effect was found significant, linear contrasts were generated to compare daily measurements to a reference value (i.e., average value of measurements on D-6 and D-5 – thermoneutral condition). In all cases, pig was the experimental unit and differences between means were tested by PDIFF with the Tukey adjustment. All statistical analyses were conducted using SAS software (*SAS Institute* Inc., Cary, NC, USA).

## Results

### Effects of hydration level and ambient temperature on feed and water intake

**Figure 1:**
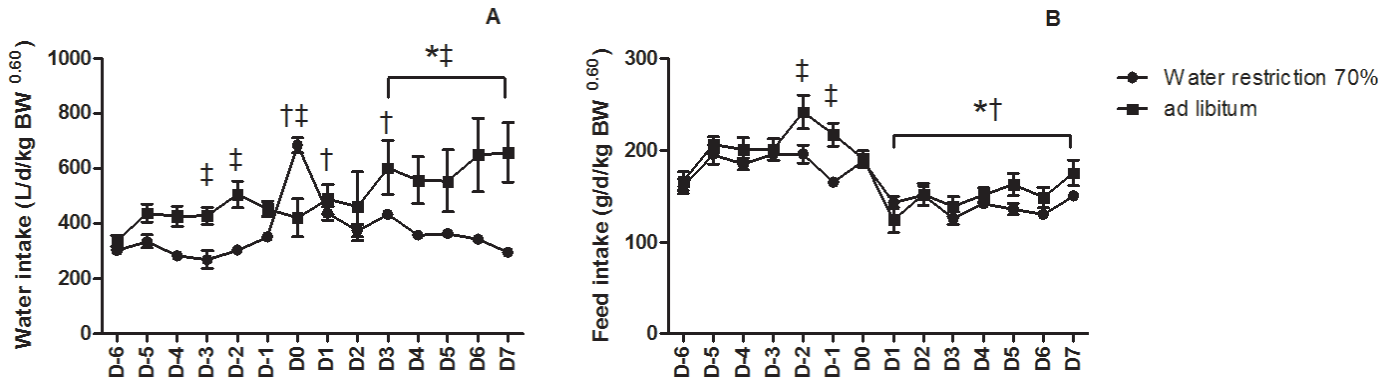
Effect of exposure to 32°C and water restriction on average daily feed and water intake. Mean ± standard error. For each water restriction treatment each point represents the mean of 6 pigs. * means that daily value is significantly different (*P<*0.05) from values at 24°C within the *ad libitum* group. † means that daily value is significantly different (*P<*0.05) from values at 24°C within the water restricted group. ‡ means that daily value was significantly affected (P<0.05) by water restriction treatment (i.e., significant daily value difference between the *ad libitum* group compared to the water restricted group).

Except for feed intake (g/d/kg BW^0.60^), the interaction between hydration level and ambient temperature was not significant for the other parameters. Accordingly, the results related to the hydration and temperature levels are presented separately in Table 1. Details on the interaction between hydration level and days of exposure are presented in Figure 1.

**Table 1:** Effects of water restriction and ambient temperature on average daily water and feed intake (mean ± standard error).

	Water restriction		Temperature		RSD^1^	*P* values^2^				
	70%	AL	24°C	32°C		W	T	W×T	D	W×D
**Number of pigs**	6	6	12	12						
**Average BW (kg)**	34.7±3.6	37.0±3.4	33.7±2.8	38.0±3.2	2.9	0.7160	0.0025	0.9587	-	-
**Water intake (L/d)**	3.10±1.01	4.42±1.96	3.06±0.86	4.29±1.91	1.04	0.0310	0.0001	0.3474	0.2856	0.0001
**Water intake (L/d/kg BW^0.60^)**	0.37±0.20	0.50±0.20	0.37±0.01	0.48±0.20	0.11	0.0267	0.0001	0.7055	0.1756	0.0001
**Feed intake (kg/d)**	1.36±0.11	1.55±0.35	1.61±0.28	1.34±0.28	150	0.1160	0.0001	0.0751	0.0001	0.0010
**Feed intake (g/d/kg BW^0.60^)**	162±30	177±42	195±33	151±29	18	0.1664	0.0001	0.0425	0.0001	0.0017

1 Residual standard deviation of the model.

2 From an ANOVA with a linear mixed model including the effect of water restriction (W), ambient temperature (T), day of experiment (D) and interactions as fixed effects.

As expected water intake expressed in L/d was 30% lower on average in the restricted group than in the control (*ad libitum*) group. This difference was slightly reduced when water intake was corrected for the metabolic body weight (i.e., −26% on average). Further, water intake significantly increased at 32°C (i.e., +30% on average) (Table 1). The kinetics of daily water over the experimental period is presented in Figure 1A. As expected, water intake for the restricted treatment was “numerically” lower when compared to the *ad libitum* treatment, excepted on D0. According to the Tukey's multiple comparison tests, the hydration level effect was significantly different between the two groups on D-3, D-2, D0, D3, D4, D5, D6 and D7 and tended to be different at D-4 (P = 0.064) (Figure 1A).

Regarding feed intake, water restriction did not significantly influence daily feed intake on the overall experimental period (Table 1). However, the *ad libitum* pigs had a higher feed intake compared to water restricted pigs on D-2 and D-1. In addition, as expected, both groups significantly reduced their feed intake at 32°C (i.e., from D1 to D7) compared to 24°C (i.e., −22% on average). Therefore under thermoneutral conditions, voluntary feed intake is influenced by water restriction, while feed intake is influenced by temperature under heat stress exposure (Figure 1B).

### Effects of hydration level and ambient temperature on thermoregulatory responses

**Figure 2:**
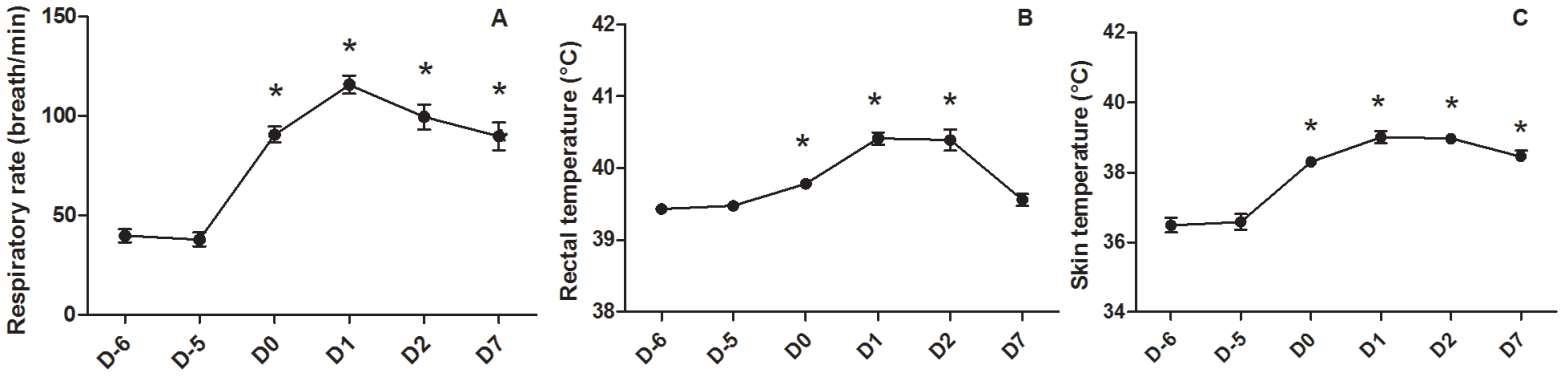
Effect of exposure to 32°C on physiological parameters in growing pigs. Mean ± standard error. * means that daily value is significantly different (*P<*0.05) from values at 24°C (i.e., mean of D-6 and D-5).

No interaction between hydration level and ambient temperature was found for physiological parameters related to the thermoregulatory response (i.e., respiratory rate, rectal temperature and skin temperature). Therefore, findings related to the hydration and the temperature levels are presented separately in Table 2. Details on the evolution of physiological parameters during the experimental period are presented in Figure 2.

**Table 2:** Effects of water restriction and ambient temperature on physiological, hematological and hemorheological parameters (mean ± standard error).

	Water restriction		Temperature		RSD^1^	*P* values^2^				
	70%	AL	24°C	32°C		W	T	W×T	D	W×D
**Number of pigs**	6	6	12	12						
**Number of measurements**	36	36	24	48						
**Respiratory rate (breath/min)**	88±17	89±18	39±0	113±7	17	0.6364	0.0001	0.4108	0.0063	0.4313
**Rectal temperature (°C)**	40.0±0.2	40.0±0.2	39.4±0.0	40.3±0.1	0.2	0.8536	0.0001	0.2693	0.0001	0.6633
**Skin temperature (°C)**	37.9±0.5	38.1±0.5	36.5±0.2	38.7±0.2	0.6	0.4048	0.0001	0.6694	0.0143	0.8179
**White blood cells (10^9^/L)**	17.2±0.5	18.9±0.4	17.5±0.1	18.3±0.4	2.0	0.3177	0.1347	0.1936	0.0136	0.4632
**Red blood cells (10^12^/L)**	6.92±0.07	6.74±0.04	6.69±0.01	6.90±0.02	0.30	0.3165	0.0261	0.2200	0.6980	0.7587
**Hemoglobin (g/dL)**	11.8±0.1	11.9±0.1	11.6±0.0	11.9±0.0	0.5	0.5336	0.0314	0.1787	0.6687	0.8704
**Mean cell volume (fL)**	56.3±0.2	57.5±0.2	57.2±0.0	56.7±0.1	0.8	0.3733	0.0396	0.6884	0.0552	0.6728
**Mean cell hemoglobin concentration (g/dL)**	30.3±0.1	30.6±0.1	30.2±0.0	30.5±0.0	0.5	0.1329	0.0391	0.8895	0.8201	0.5149
**Platelets (10^9^/L)**	416±25	402±16	449±6	388±11	46.5	0.5137	0.0004	0.6857	0.0014	0.2182
**Reticulocytes (%)**	1.62±0.29	2.05±0.37	1.40±0.01	2.04±0.27	0.64	0.0810	0.0049	0.9817	0.0001	0.8218
**Hematocrit (%)**	36.0±0.2	36.3±0.3	35.4±0.1	36.5±0.14	1.5	0.2855	0.0220	0.9093	0.0207	0.9689
**Blood viscosity (mPa/s)**	6.6±0.2	6.7±0.1	6.9±0.1	6.5±0.09	0.5	0.2678	0.0416	0.6960	0.0549	0.7391
**RBC deformability at 3 Pa (u.a)**	0.40±0.00	0.40±0.00	0.40±0.00	0.40±0.00	0.02	0.6884	0.9884	0.1378	0.2804	0.6435
**RBC deformability at 30 Pa (u.a)**	0.52±0.00	0.52±0.00	0.51±0.00	0.52±0.00	0.01	0.5973	0.0284	0.5019	0.1462	0.6979
**RBC aggregation (%)**	59.1±1.0	61.1±1.3	59.2±0.2	60.6±1.0	2.7	0.2357	0.0846	0.5966	0.0001	0.2021
**RBC disaggregation threshold (s^−1^)**	105±3	118±7	104±1	116±3	82	0.0118	0.1695	0.2743	0.0024	0.1190

RBC = red blood cell.

1 Residual standard deviation of the model.

2 From an ANOVA with a linear mixed model including the effect of water restriction (W), ambient temperature (T), day of experiment (D) and interactions as fixed effects.

Water restriction had no effect on the physiological parameters: i.e., respiratory rate, rectal temperature and skin temperature. Conversely, exposure to heat stress (i.e., mean of D-6 and D-5 versus mean of D0, D1, D2 and D7) caused a significant increase in these three physiological parameters (Table 2). Regarding the time-course of physiological parameters, respiratory rate (Figure 2A) and skin temperature (2C) increased significantly after heat stress exposure (i.e., D0, D1, D2 and D7) compared to the thermoneutral condition (i.e., D-6 and D-5). The same pattern was observed for rectal temperature except that D7 was not significantly different compared to the thermoneutral condition (Figure 2B). The maximum difference was observed at D1 for the three physiological parameters (Figure 2). Interestingly, we observed a significant reduction of the respiratory rate on D2 (p = 0.028) and D7 (p<0.001) compared to D1 (Figure 2A). Further, we observed a significant reduction of rectal temperature (p<0.001) and skin temperature (p = 0.022) on D7 compared to D1 (Figure 2B and 2C) suggesting successful physiological acclimation to heat stress.

### Effects of hydration level and ambient temperature on hematological and hemorheological parameters

**Figure 3:**
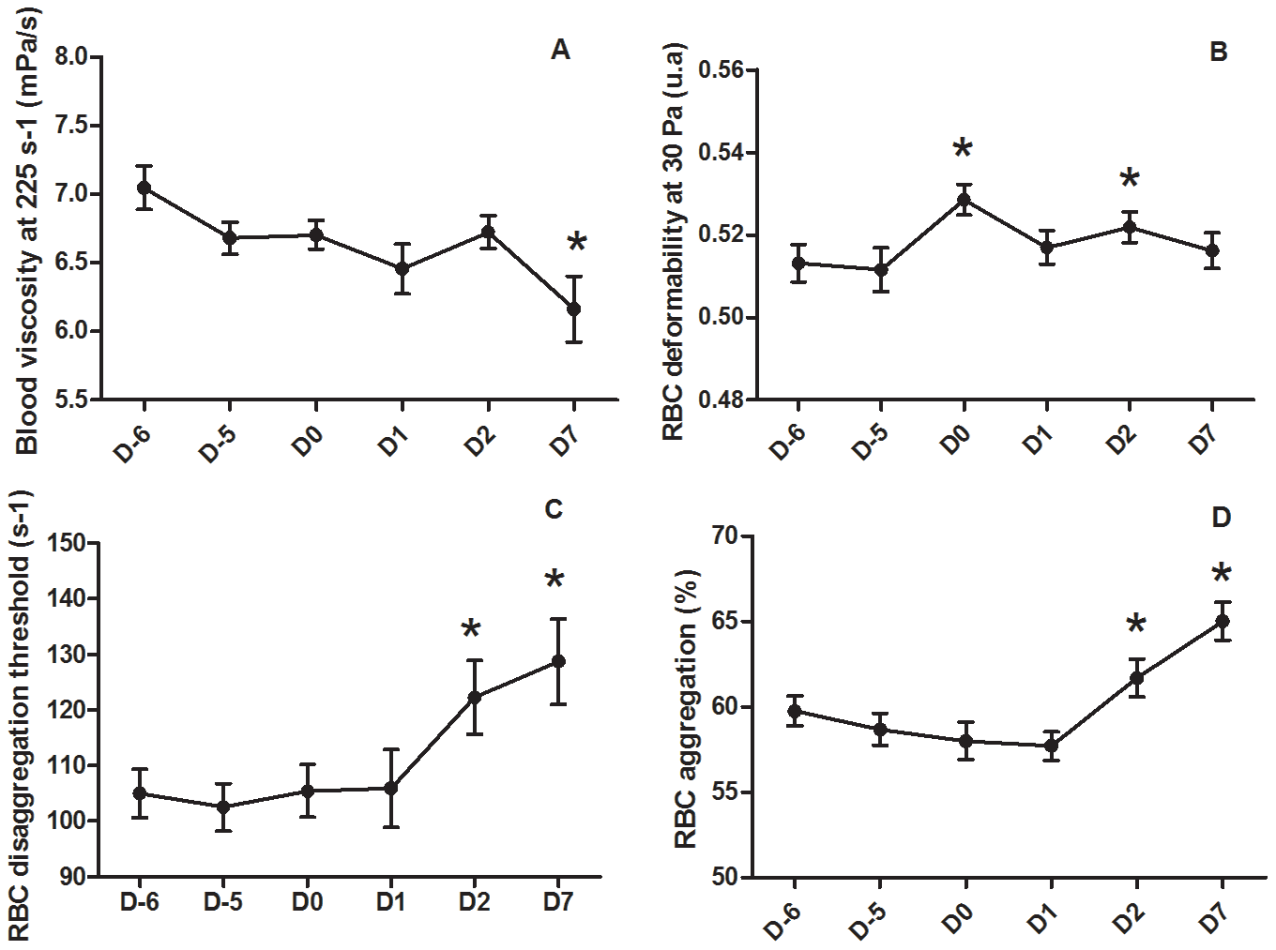
Effect of exposure to 32°C on hemorheological properties in growing pigs. Mean ± standard error. * means that daily value is significantly different (P*<*0.05) from values at 24°C (i.e., mean of D-6 and D-5).

**Figure 4:**
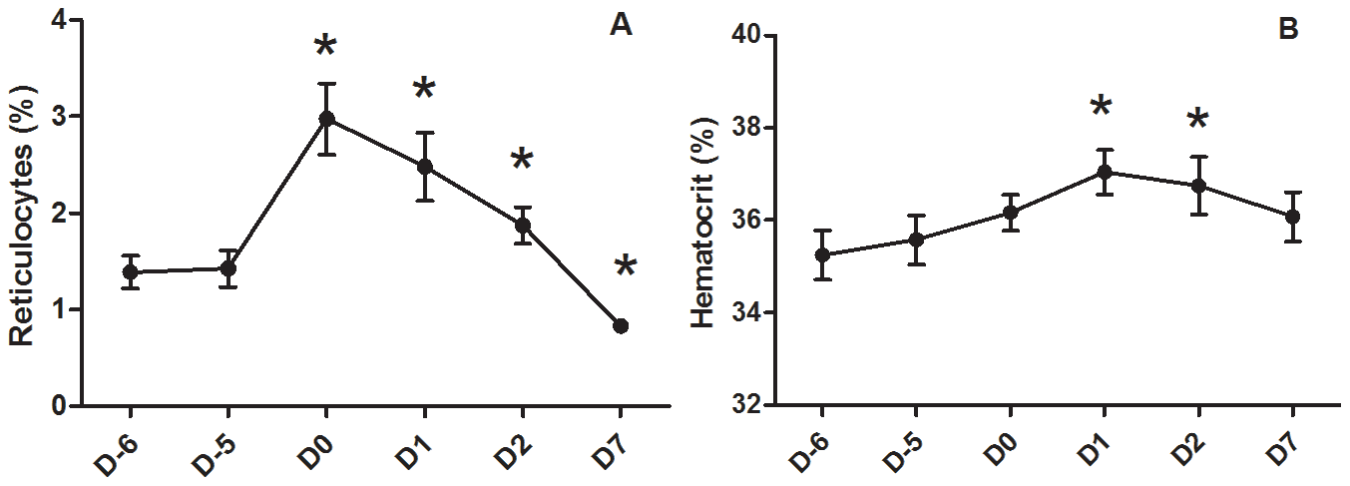
Effect of exposure to 32°C on percentage of reticulocytes and hematocrit in growing pigs. Mean ± standard error. * means that daily value is significantly different (P*<*0.05) from values at 24°C (i.e., mean of D-6 and D-5).

The effect of hydration level, ambient temperature, and interaction on hematological and hemorheological parameters are summarized in Table 2. Details on the evolution of hematological and hemorheological parameters during the experimental period are presented in Figures 3 and 4.

We observed no interaction effect between hydration level and ambient temperature on hematological and hemorheological parameters. Moreover, although the percentage of reticulocytes tended to be reduced in the water restricted group compared to the *ad libitum* group (p = 0.081), we observed no effect of hydration level on hematological and hemorheological parameters. Red blood cells (RBCs) count, hemoglobin concentration, mean cell hemoglobin concentration, percentage of reticulocytes, hematocrit and RBC deformability at 30 Pa were higher under heat stress compared to the thermoneutral period. Mean cell volume, platelets count and blood viscosity were lower during heat stress than at 24°C. RBC aggregation tended to be higher during heat stress exposure compared to the thermoneutral period (p = 0.085). We observed no difference for white blood cells count, RBC deformability at 3 Pa and for the RBC disaggregation threshold (Table 2) between the thermoneutral and the heat stress periods.

The changes in blood viscosity over time tended to be significant (time effect: p = 0.055). The contrast analysis revealed a reduction of blood viscosity on D7 compared to the thermoneutral condition (Figure 3A). We observed no time effect for RBC deformability at 30 Pa (p = 0.146) even if the contrast analysis revealed a slight and transient increase of RBC deformability measured at 30 Pa on D0 and D2 compared to the thermoneutral period (Figure 3B). Regarding RBC disaggregation threshold and aggregation time-course, we observed a significant increase on D2 and D7 compared to the thermoneutral period (Figure 3C and 3D, respectively). Interestingly, compared to the thermoneutral period, the percentage of reticulocytes increased significantly at D0 (i.e., first day of progressive heat stress exposure), and then decreased at D1 (p = 0.02), D2 (p<0.001) and D7 (p<0.001) compared to D0. At D7, the percentage of reticulocytes was lower than the baseline level (Figure 4A). We observed a slight increase of hematocrit on D1 and D2 compared to the thermoneutral period (time effect: p = 0.021) (Figure 4B). In addition, contrast analysis revealed that hematocrit significantly decreased between D1 compared to D7 (p = 0.035).

## Discussion

The present study showed that while water restriction had no effect on physiological and biological parameters in Large White pigs, important physiological and hematological/hemorheological changes occurred during the thermal acclimation period at 32°C.

Surprisingly, water restriction did not exacerbate the effect of thermal heat stress on physiological, hematological and hemorheological responses. We suspect that water restriction was not large enough to really challenge the thermoregulatory responses of pigs. Moreover, it could be hypothesized that higher water intake on D0 (i.e., when heat stress exposure began) in water restricted pigs would have completely modified their hydration status (i.e., reconstitution of the amount of water stored in the body). This could explain the lack of significant effect of water restriction thereafter. Pigs in the restricted group were accustomed to drink a large amount of water at 09:00 (i.e., because of the water restriction) whereas *ad libitum* pigs drunk water more regularly during the diurnal period. When ambient temperature rises, pigs usually reduce their physical activity by limiting the time spent in standing/sitting positions. These behavioral changes are considered as an adaptation to reduce heat production and/or increase heat dissipation but have subsequent negative consequences on feeding and drinking activities. From that, we suggest that the greater water intake in the restricted than in the *ad libitum* pigs on D0 could be explained both by their large water intake prior to the beginning of thermal stress (i.e., at 10:00) and by the behavioral changes of the *ad libitum* group under heat stress exposure.

In pigs, it is generally accepted that water restriction results in a reduced voluntary feed intake. In the present study, this assumption was confirmed at 24°C. We observed a similar reduction of feed intake in the two groups at 32°C, which was expected as this is a common response among species during heat stress exposure [2], [11], [12]. This reduced feed intake may be a strategy to minimize metabolic heat production and may explain, in part, the physiological adaptations (i.e., reduction of respiratory rate, rectal and skin temperatures) that we observed in the present study. The lack of difference between the two groups for feed intake under heat stress could be explained by the above mentioned behavioral changes and/or by the fact that heat stress is a more important factor affecting feed intake than mild water restriction.

To our best knowledge, this study is the first one to observe an acute increase of the percentage of reticulocytes after heat stress exposure in growing pigs. It has been shown that heat stress increase skin blood flow circulation to promote heat loss [13]. This blood redistribution to the skin causes a reduction of blood flow to the other tissues, such as the intestinal epithelium, which may in turn cause tissue hypoxia [2], [13]. Therefore, the acute increase of the percentage of reticulocytes could be caused by an acute release of reticulocytes by the bone marrow in order to increase hemoglobin mass and to protect several tissues from hypoxia. The slight but significant increase of hematocrit under heat stress could be caused, at least partly, by this transient rise of the percentage of reticulocytes. This hypothesis is supported by the fact that the time course of the percentage of reticulocytes had the same time-course than physiological parameters (i.e., rectal temperature, respiratory rate and skin temperature) reflecting the stress caused by heat exposure. Moreover, we observed a positive and significant correlation (Pearson coefficient) between the percentage of reticulocytes and the respiratory rate (r = 0.34; p<0.05 – data not shown) or skin temperature (r = 0.30; p<0.05 – data not shown).

We also observed a delayed increase in RBC aggregation and RBC disaggregation threshold (i.e., the minimal strength needed to fully separate RBC aggregates) on D2 and D7 after heat exposure. RBC aggregation properties depend on plasma factors, such as fibrinogen level, and cellular factors (i.e., RBC aggregability) [14], [15]. We did not measure plasma proteins concentration or RBC aggregability properties. Further studies are needed to understand the delayed increase in RBC aggregation after heat stress exposure that we observed in the present study.

The increased RBC deformability during heat stress period is surprising since reticulocytosis is usually accompanied by a reduction of RBC deformability because the membrane of reticulocytes is more rigid than the membrane of mature RBCs. In addition, both the slight increase in mean cell hemoglobin concentration and decrease of mean cell volume suggest RBC dehydration during heat stress exposure, which should cause a rigidification of RBCs. However, several hormonal changes may occur during heat stress in growing pigs and one of them is the increase of plasma insulin concentration [16]. Insulin is able to stimulate the production of nitric oxide by the red blood cells through the PI3-kinase/Akt kinase pathway, which may increase RBC deformability through S-Nitrosylation of several RBC cytoskeleton proteins [17]. Whether this mechanism was present in our study requires further works.

The slight but significant decrease of blood viscosity at D7 of heat exposure despite the increase of hematocrit at the beginning of heat stress is very surprising too. Since the mean value of RBC deformability during heat stress period was increased in comparison to values measured at 24°C, one may suggest that this increase could have partly offset the consequences of the transient rise of hematocrit on blood viscosity. However, the time-course of RBC deformability does not fully support this hypothesis. The reduction of blood viscosity and the slight transient increase of RBC deformability during heat exposure could lead to a reduction of peripheral resistance, hence facilitating tissue oxygenation and blood supply to heat exchange surfaces [18]. These changes could thereby participate in the acclimation response observed in the present study.

In conclusion, this study showed that one week of heat stress exposure causes significant acclimation responses in Large White pigs. These physiological acute adaptations to heat stress could be explained by a reduction of feed intake, a reduction in blood viscosity, an increase of red blood cell deformability and an important acute release of reticulocytes to limit tissue hypoxia.
